# Zoonotic infections in semi-domesticated Eurasian tundra reindeer (*Rangifer tarandus tarandus*) in Fennoscandia – a review

**DOI:** 10.1186/s42522-025-00140-0

**Published:** 2025-04-08

**Authors:** Morten Tryland, Kayla Joy Buhler

**Affiliations:** Department of Forestry and Wildlife Management, University of Inland Norway, Koppang, N-2480 Norway

**Keywords:** Bacterium, Food, One health, Reindeer husbandry, Parasite, Virus, Wildlife, Zoonoses

## Abstract

Eurasian tundra reindeer (*Rangifer tarandus tarandus*) make up the basis for reindeer herding in Norway, Sweden and Finland, hosting about 640 000 animals. The animals are mostly free-ranging, with the exception of a few seasonal gatherings. Loss and fragmentation of pastures due to other types of land use, together with climate change and even conservation of predators, are challenging reindeer herding, leading to recent mitigations such as increased feeding. Whereas the average Norwegian consumes about 300 gr/person/year of reindeer meat, Sweden about 100 gr/person/year and Finland about 400 gr/person/year, reindeer meat and products constitutes a much larger part of the diet to members of herding communities. Preparing reindeer meat with no thorough heat treatment (e.g., drying, smoking, curing or raw consumption) can be found in many arctic and sub-arctic people’s cultures, and interest for reindeer meat that is not heat-treated has also grown (e.g., carpaccio-style), which can dramatically impact pathogen transmission. There is a wide range of zoonotic parasites, bacteria and viruses that potentially can be transferred from reindeer to human, but it can be concluded that the zoonotic threat from close contact with reindeer or the consumption of reindeer meat and products thereof currently is of restricted magnitude. However, due to the challenges that reindeer populations are facing and the mitigation by increased feeding, the infection biology of zoonotic infections may change and we may face emerging diseases (i.e., pathogens new to the host and region), such as the recently appearing prion disease, Chronic wasting disease (CWD), and re-emerging diseases, such as the alimentary form of necrobacillosis.

## Introduction

*Rangifer tarandus* is a species in the Cervidae (deer) family of ruminants with a circumpolar distribution, commonly called reindeer in Eurasia and caribou in North America, or just *Rangifer* [1]. *Rangifer* includes seven subspecies, such as the Eurasian tundra reindeer (*Rangifer tarandus tarandus*) in Fennoscandia and several caribou subspecies in North America [2]. Reindeer herding is practised by about 100,000 people belonging to roughly 30 ethnic and Arctic peoples, all implementing different kinds of reindeer husbandry as a core component of their culture and livelihood [3].

Reindeer herders are the pastoralists of the north, rearing animals for consumption and trade. Sami reindeer herding is organized through social networks, often between close relatives. These herding units are called Siida (North Sámi) in Norway and Sami reindeer herding village (Sameby) in Sweden. With a few exceptions (Tamreinlagene in Norway and Concession villages in Sweden), only Sami people have the right to pursue reindeer herding. This contrasts with the situation in Finland, where any EU citizen living in the 56 designated reindeer herding districts (paliskunta) in the provinces of Lappland and Oulu has the right to own reindeer. Seasonal availability of forage and water requires movement of animals and people across the landscape. Reindeer herding has traditionally been conducted in a semi-nomadic manner, with transitions between summer and winter pastures [4].

Animals are free ranging for most of the year but are usually gathered a few times for ear-tagging calves for ownership, selection of slaughter animals, castration of some bulls, anti-parasite treatment (if practiced), and seasonal transition between pasture areas [4]. Reindeer herding was traditionally conducted with the assistance of herding dogs by foot, but motorized vehicles (e.g., snowmobiles, motorbikes, 4-wheelers and helicopters) are now commonly used to herd and gather animals. For the purpose of this review, reindeer herding will describe direct work with reindeer, while reindeer husbandry describes wider aspects, including practical work with reindeer, hunting, and the transformation of reindeer herding towards an economically and biologically sustainable industry.

In Fennoscandia (for simplicity defined as Norway, Sweden and Finland in this review), reindeer herding involves around 640,000 animals. Reindeer herding is conducted on about 40%, 50% and 33% of the land of Norway, Sweden and Finland, respectively (Table 1).

**Table 1:** Key characteristics of reindeer herding in Norway, Sweden and Finland

Country	Pasture area of land mass	Reindeer owners	Reindeer	Slaughtered Animals^1^	Tons of meat
Norway [5]	40%	3 300	217 809	70 533	1 600
Sweden [6]	50%	4 600	240 314	40 528	1 025
Finland [7]	33%	4 300	185 356	70 387	1 800
Total		12 200	643 479	181 448	4 425

The table shows the land area used as reindeer pasture, the number of reindeer owners and semi-domesticated reindeer, the number of slaughtered reindeer and tons of meat produced during the reindeer herding year 2021–2022 in Norway, Sweden and Finland

^1^ Animals slaughtered and health marked at approved slaughterhouses and game handling establishments

Reindeer herding has been able to adapt to changes over time, such as the conservation of predators like the wolverine (*Gulo gulo*), lynx (*Lynx lynx*) and golden eagle (*Aquila chrysaetos*) [5]. However, several societal and ecological shifts are increasingly challenging reindeer herding. Loss of pastures due to other types of land use, such as powerplants, windmills, roads, cabins, mining, and forestry, is likely the biggest threat to traditional reindeer herding today [5]. Climate change is another threat that influences the seasonality of pasture resources and may create ice-locked winter pastures and other unpredictable environmental conditions, which again increases pressure for alternative land-use strategies [6]. About 12,200 people are registered as reindeer owners in Norway, Sweden, and Finland (Table 1) [7–9].

Reindeer meat is mainly associated with the Sámi culture and heritage, representing a source of meat produced on pasture resources that are unsuitable for most other species. In Norway, about 1,600 tons of reindeer meat are sold via registered slaughterhouses (Table 1) and about 300 tons are consumed by reindeer herders and local markets [10]. The average consumer in Norway eats about 300 g of reindeer meat per year and consumption is three times higher in the northern part of the country (Nordland, Troms, and Finnmark Counties) [10]. In Sweden, the production of reindeer meat corresponds to a consumption of about 100 g/person/year [11]. However, since most of the meat is sold on a local scale or to restaurants and catering, only 20–30% of the reindeer meat is available in regular grocery stores, most of it offered as packages of frozen, sliced meat (“skav”) [12]. About 400 g/person/year of reindeer meat is consumed in Finland [13], down from about 500 g/person/year in 2010 [14].

Reindeer meat is considered healthy, as it is a lean meat source with only 2% lipid content and is also a source of omega-3 fatty acids and essential vitamins and minerals [15]. Furthermore, the levels of heavy metals and persistent organic pollutants (POPs) are low compared to other sources [16, 17] and the levels and effects of radioactive fallouts following the accident at the Chernobyl nuclear power plant (Ukrainian Soviet Socialist Republic, 1986) seem today to be restricted [18].

Predator pressure, loss and fragmentation of pastureland and climate change together represents restrictions on how traditional reindeer husbandry can be conducted. One mitigation strategy is supplementary feeding, which has increasingly been introduced throughout the reindeer herding regions in Fennoscandia [19]. The practice of feeding, especially when conducted in corrals at full rations and over longer periods of time, makes reindeer herding more dependent on agriculture (such as the production of feedstuff) and creates closer interactions between animals and people through handling and feeding. This may impact diseases that can be transmitted between animals during feeding, such as diarrhoea, rumen acidosis and bloat [d 20], but it could also influence diseases that are associated with stress, close-contact, and hygienic conditions at feeding spots [21]. In turn, this could influence the transmission of zoonotic pathogens that circulate in semi-domesticated reindeer herds in Fennoscandia.

The aim of this review was to gather and discuss existing knowledge on zoonotic pathogens that are directly transmitted from reindeer and their importance in a One Health perspective, acknowledging that the environment, human health, and animal health are linked and interdependent.

## Reindeer health and zoonotic infections

Although some reindeer are slaughtered in the field for the herder´s own consumption or in remote areas by using mobile slaughter facilities (specialized trucks or buses), most of the reindeer are processed in centralized slaughterhouses that are approved for farmed game (reindeer).

Due to long distances to slaughterhouses, transport is usually necessary. This is accompanied by detailed national and EU regulations and restrictions for transport time, space, and availability of water and food. Gathering reindeer from mountain pastures, handling, corralling, transporting, and corralling again at the slaughterhouse may contribute to poor animal welfare. In addition, stress may result in abnormal odour and quality from the slaughter carcass, which may lead to condemnation of the carcass [22]. Stress is associated with increased levels of circulating stress hormones, such as glucocorticosteroids, which have immunosuppressive effects [23] and may facilitate diseases and spread of infectious agents among reindeer [21].

As part of the slaughter process, reindeer are subjected to a veterinary *ante mortem* inspection (AMI) to secure good animal welfare and to ensure that no animals are entering the slaughter line with diseases or conditions that can be harmful for slaughterhouse workers and consumers. After dressing, reindeer carcasses with internal organs intact are subjected to a *post mortem* inspection (PMI) under the responsibility of a veterinarian that is associated with the responsible authority for the country; Norwegian Food Safety Authority (NFSA), Swedish Food Agency (SFA) and Finnish Food Authority (FFA). The main aim of the AMI and PMI is to protect human health, animal health and animal welfare [24]. On suspicion, detailed diagnostic work and pathogen verification may be conducted to identify notifiable diseases and to implement mitigation strategies against and spread of transmissible diseases.

Whereas emaciation was the most common cause of rejection for slaughter, trauma and parasites were reported as the most common findings during meat inspection in a review from slaughterhouses in Finnmark County, Norway, during 2004–2010 [25]. Trauma is often caused by herding, corraling, handling, and transport and can lead to affected parts of the animal being discarded due to bone fractures and subcutaneous/soft tissue bleedings. Inspection for parasites often focuses on subcutaneous warble fly larvae (*Hypoderma tarandus*), muscle cysts from *Sarcocystis* sp., brain worm (*Elaphostrongylus rangiferi*) or lung pathology due to larval development (brain worm and others), and *Echinococcus granulosus* cysts, none of which are directly transmissible to humans [25]. Other findings, such as peritonitis, pleuritis, abscesses, and tumours, may lead to total or partial local condemnation but are often not diagnosed with the aim of identifying a potential causative infectious agent.

In Sweden, the PMI findings are usually reported in gross categories, such as parasites, trauma, emaciation, inflammatory processes, and poor slaughter hygiene [26], with no identification or verification of potential causative infectious agents when infections are identified. The most common findings in Sweden during 2015–2016, covering approximately 110,000 animals, were non-zoonotic parasites. Inflammatory processes and trauma were low in prevalence and there were no indications of food safety hazards, including zoonoses or ongoing reindeer epizooties [26].

In Finland, a study addressing the effect of transport (*n* = 669 738) to slaughterhouse on the wellbeing of reindeer (*n* = 669 738), summarized the most common indicators of compromised welfare at PMI to be bruises and fractures, aspiration of rumen content and abnormal odour [22]. Notifiable diseases, which may differ between countries and regions, are reported (when verified) to responsible authorities.

**Fig. 1:**
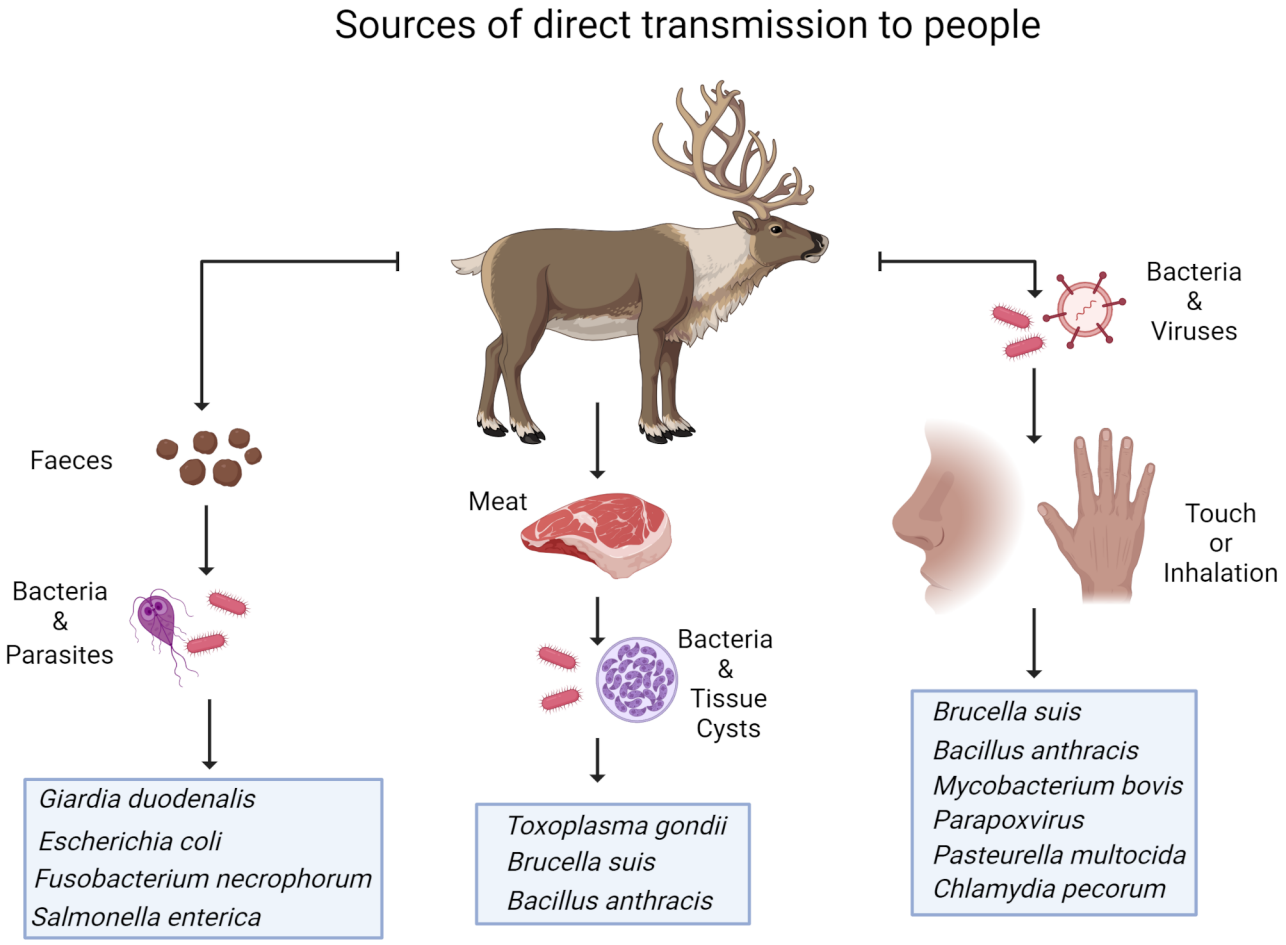
Modes of transmission of pathogens from reindeer to humans

Zoonoses are infectious diseases that are naturally transmissible from vertebrate animals to humans (World Health Organization, WHO, 2020). Pathogens may be transmitted from reindeer to humans through direct contact (touch or inhalation), contact with faeces or via consumption of reindeer meat and products (Fig. 1). Reindeer are not known to be the main host or reservoir for hazardous zoonotic pathogens in Fennoscandia. However, in a bacteriological investigation of faecal samples from 470 reindeer calves (October, 6–7 months of age) from Finland (*n* = 410) and Norway (Finnmark, *n* = 60), *Listeria monocytogenes* (3%) and *Yersinia* spp. (10%; mainly *Y. kristensenii*, but also *Y. enterocolitica*) were cultivated and identified [27]. Furthermore, Shiga toxin genes (stx-1 and/or stx-2; 25%) were detected by PCR, indicating the presence of Shiga toxin producing *Escherichia coli* (STEC) [27]. These findings indicate that potential food-borne bacterial pathogens are present in the gastrointestinal tract of reindeer, calling for good slaughter hygiene to keep them from entering the food chain.

Thus, given the right circumstances, several pathogens, through contact or consumption, may be transmitted from reindeer to humans. While omitting infectious agents that are typically associated with poor meat hygiene and storage conditions, we will briefly discuss parasites, bacteria, viruses and prions that affect reindeer health and that may represent a zoonotic risk to humans, defining risk as “the probability of harm and the severity of that harm” [28].

### Parasitic zoonoses

#### Toxoplasmosis (*Toxoplasma gondii*)

*Toxoplasma gondii* is a protozoan parasite that infects most species of warm-blooded animals, including humans, and causes the disease toxoplasmosis. The definitive hosts for *T. gondii* are members of the family *Felidae* (cats and wild relatives) which shed oocysts in the environments. *Toxoplasma gondii* usually causes subclinical infections in immunocompetent humans but may cross the placenta and cause congenital abnormalities and encephalitis, especially when the mother is exposed for the first time during pregnancy [29]. Postnatal human infections can occur through the ingestion of oocysts, often via soil/cat faeces, or via consumption of bradyzoites, a parasite stage present in meat from a wide range of animals that are intermediate hosts [30, 31]. Antibodies against *T. gondii* have been detected in most herds of caribou in North America [32, 33], while a seroprevalence of about 1–2% has been found in semi-domesticated and wild reindeer in Fennoscandia [34–36]. Higher seroprevalence, exceeding 20% in some herds, has been found in reindeer with a higher degree of domestication [34].

There is no evidence that people have contracted toxoplasmosis associated with the intake of caribou or reindeer meat. However, a study conducted among 267 residents of Cree communities in James Bay, Canada, revealed a seroprevalence of 9% [37]. Furthermore, in Nunavik, Canada, where most of the Inuit population has antibodies against *T. gondii* [38], studies have indicated a seroprevalence of 18–26% in caribou. However, no *T. gondii* specific DNA could be detected in tissues of the seropositive animals [39, 40]. These studies may indicate that caribou, and possibly reindeer in Fennoscandia, are not major sources of infection to humans. Human exposure to *T. gondii* in Fennoscandia is not well studied, but antibodies are common in parts of Sweden (23% in Uppsala) [41] and Norway (8.3% in former Buskerud County and 10.4% in former Sør-Trøndelag County) [42]. However, inhabitants of these regions are not largely associated with reindeer herding and extensive consumption of reindeer meat, suggesting other sources of infection.

#### Giardiasis (*Giardia duodenalis*)

*Giardia duodenalis* is a flagellated protozoan parasite commonly found in mammals. It colonizes the small intestine and may cause severe diarrhoea in animals. It is one of the main causes of diarrhoea in children. An investigation of 155 wild reindeer in Norway revealed a prevalence of 7% for *G. duodenalis* [43]. A recent investigation (immunofluorescence and/or PCR) of faeces from semi-domesticated reindeer in two herds in Finnmark County, Norway, reported a prevalence of 10.9% in Spierttagáisá (in 5 calves, 0 adults, *n* = 46) and 1.5% in Neiden (1 calf, 0 adults, *n* = 68) [44]. All PCR generated sequences belonged to assemblage A (sub-assemblage AI), which is known to have zoonotic potential but has not previously been reported from semi-domesticated reindeer [44]. There is no evidence that *G. duodenalis* has pathogenic potential in reindeer [45, 46] and there is no evidence of transmission from reindeer to humans.

#### Cystic echinococcosis (*Echinococcus granulosus*)

**Fig. 2:**
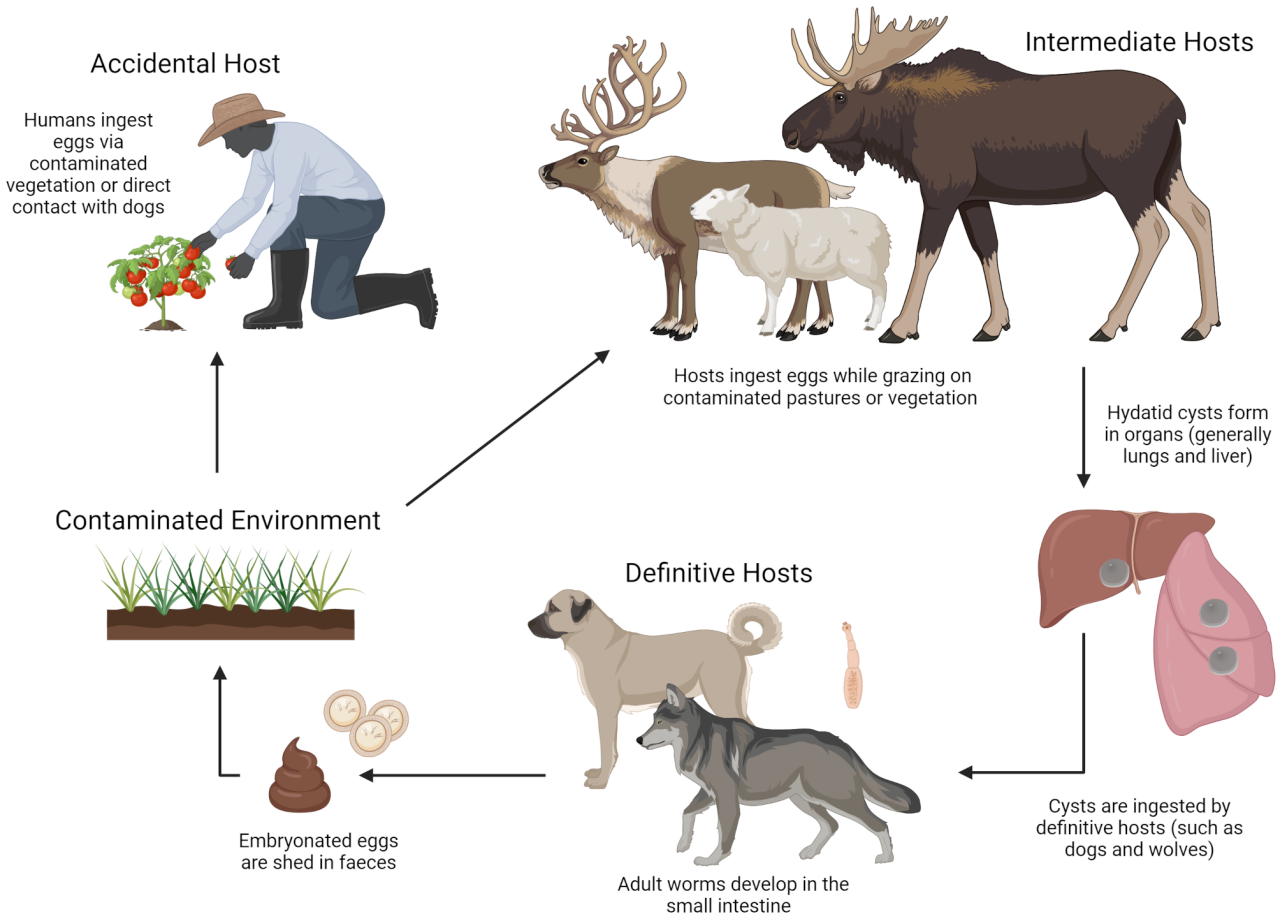
The life cycle of the tape worm *Echinococcus granulosus*. The parasite is not transmitted directly from reindeer to humans – both reindeer and humans are intermediate hosts. Parasite cysts may be detected in lungs and liver during slaughter

*Echinococcus granulosus* (*Taenidae* family, genus *Echinococcus*) is a 5–6 mm long tapeworm that lives in the small intestine of dogs and wild Canids, such as wolves (*Canis lupus*) (Fig. 2). Reindeer are intermediate hosts and are infected by ingesting parasite eggs from canid faeces. Larvae spread from the intestines into many different tissues, but they are most commonly found in the lungs, where cysts develop over many years containing numerous new offspring, called protoscoleces [46]. The cyst can measure 14–15 cm in reindeer [47, 48], and dogs and wolves are infected by the ingestion of internal organs containing cysts.

In the same way as reindeer, humans may serve as an intermediate host for the parasite after ingestion of eggs from dog faeces. This means that the parasite is not transmitted directly from reindeer. However, we have chosen to include this parasite in this review as reindeer carcasses are routinely investigated for cysts as part of meat control. Cysts develop in various organs, but a screening from the reindeer herding regions in Finnmark County, Norway, during 1952-56 reported that 29 of 34 diagnostic cases with echinococcosis had cysts in the lungs (remaining five cases having cysts in the liver) [49]. The cysts develop continuously and may be up to 20 cm in diameter in humans [50]. Symptoms result from organ deficiencies in the lungs and liver and life-threatening anaphylactic shock may occur if cysts rupture [50].

*Echinococcus granulosus* used to be enzootic in Finnmark County, Norway, which hosts about 70% of the semi-domesticated reindeer population in Norway. A prevalence of 9.6% was found in reindeer from this region during slaughter in 1956-58 [47]. It was common practice to feed slaughter waste to herding dogs with no prior heat treatment. After a campaign that reduced access to slaughter waste for dogs and provided anthelmintic treatment, the prevalence was reduced to 0.1% in 1980–1981 [51]. The last report of this parasite in reindeer from Finnmark was documented in 2003 [46]. *Echinococcus granulosus* is a notifiable disease.

The current status for Sweden is similar to the one in Norway. The parasite is more common in Finland, especially in reindeer located near the Russian border, where a prevalence of 30% has been documented in a Finnish-Russian wolf population [52]. Mandatory thorax radiographic surveillance for tuberculosis was initiated in the Nordic countries after the second World War, which also contributed to the diagnosis of hydatid cysts of *E. granulosis*, but this practice has been discontinued [53].

*Echinococcus granulosus* G10/*E. canadensis* was recently detected in moose (*Alces alces*) in Norway (Innlandet County), which also hosts a population of wolves. The parasite is more common in wildlife in Sweden and Finland [53], and one autochthonous human case has been reported in Finland [54].

Another cestode that may occur in reindeer is *Taenia krabbei*, for which reindeer is an intermediate host and carnivores are the main host. Cysticerci, the larval stage, which is round to oval, white and 2–4 mm long, may be found in muscles and connective tissues in reindeer. However, the parasite is regarded as rare in reindeer in Fennoscandia and is not a zoonotic pathogen [46].

#### Human myiasis and the reindeer warble fly (*Hypoderma tarandi*)

While *Rangifer* species are the definitive hosts for the reindeer warble fly (*Hypoderma tarandi*), the insect can occasionally deposit its eggs on hair of humans. The reindeer warble fly is widely distributed among semi-domesticated reindeer in Fennoscandia. Mating and oviposition take place in July and August under favourable conditions like warm weather (> 13–15 °C) and little wind [55]. The fly glues its eggs to the stem of thin summer hair. The eggs hatch after a few days and the larvae (about 0.6 mm long) penetrate and develop under the skin, causing a condition called myiasis. The larvae are encapsulated in connective tissue and fluids during winter and establish a breathing hole through the skin. In May-June, the larvae (now about 2.5–3 cm long) leave their host through the breathing hole and drop to the ground where they burrow and pupate. After a few weeks, adult flies appear and starts mating and egg laying activity [56]. Myiasis in reindeer is painful for the animals and reduces the quality of the pelt, causing economical loss for reindeer herders.

For most of the reported human myiasis cases, the larvae have invaded the eye globe (ophthalmomyiasis interna; OMI), causing visual impairment in residents or visitors to northern regions of Norway and Sweden [56, 57]. In a clinical study, the most common symptoms, typically appearing in September-December, were lymphadenopathy and single or multiple dermal swellings of the head, including the periorbital region. All patients in the study (*n* = 39; 2011–2016) had specific antibodies against an enzyme excreted by the larvae. Most of the patients (*n* = 32) were children (3–12 years old) and most were successfully treated with the anthelminthic drug ivermectin [57]. Although it affects a limited number of people, OMI is a serious and disturbing disease that can be challenging to treat. People visiting reindeer pasture areas during the host-seeking period of the reindeer warble fly are advised to wear a hat or hood [57].

### Bacterial zoonoses

#### *Escherichia coli*

*Escherichia coli* is a pathogen in ruminants that increasingly has been linked with several human outbreaks of diarrhoea associated with food items, such as meat and dairy products [58]. This is a common gram-negative facultative anaerobic bacterium found in the gut of healthy animals and people. Most strains occur as harmless symbionts, though pathogenic strains exist, with virulence generally linked to the production of shiga toxins or vero cytotoxins [59]. Cattle are considered reservoirs for toxin-producing *E. coli*, but other ruminants, like sheep and deer, can contribute to shedding in the environment [60].

Pathogenicity is generally low, though it varies depending on the microbial flora of the gastrointestinal tract or immunocompetence of individual animals. Various studies have examined reindeer for toxin-producing *E. coli* in Fennoscandia, which found that they are quite rare [61, 62]. Verocytotoxic *E. coli* is also rarely found in domestic ruminants in Fennoscandia, while high prevalence of shiga-toxin producing *E. coli* (STEC) has been found in sheep and cattle in Norway, likely due to corralling and confinement in pens [63, 64]. Only a single STEC strain from 31 *E. coli* isolates from reindeer has been found in Norway [65]. Thus, reindeer do not appear to be reservoirs for toxin-producing *E. coli*, likely due to management factors like low herd density or less intensive feeding strategies. However, as supplementary feeding becomes more intensive alongside climate change and as long travelling distances to slaughterhouses create increased dietary stress, there may be increased shedding of pathogenic strains and enhanced fecal-oral transmission from semi-domesticated reindeer in the future [60].

#### Brucellosis (*Brucella suis* biovar 4)

Rangiferine brucellosis, caused by the bacterium *Brucella suis* biovar 4, is enzootic in *Rangifer* subspecies from Siberia, Canada and Alaska [66]. So far, it has not been detected in reindeer in Fennoscandia and it is a notifiable disease. Clinical signs often impact reproductive success and can include abortion, stillbirths, metritis and orchitis [67]. Infection in both sexes can lead to abscesses and enlarged joints, bursitis and lameness [66, 68]. Transmission and maintenance of *B. suis* biovar 4 in the environment is thought to be similar to bovine brucellosis, with large amounts of bacteria shed during parturition in the uterus, udder, and milk [69]. Thus, vertical transmission across the placenta may be possible, along with environmental contamination via aborted material [66]. The bacteria are environmentally resistant, persisting for years in frozen aborted material and for months in moist conditions [67].

Cases of *B. suis* biovar 4 have been detected in muskoxen and cows, indicating that there is potential crossover from Cervidae to Bovidae [69, 70]. This raised alarms regarding transmission to domestic livestock, as *Brucella abortus* causes severe economic losses for livestock farms worldwide [71]. However, bison and cattle do not appear to develop disease when exposed to *B. suis* biovar 4 [69, 72]. In contrast, humans may become infected with *B. suis* biovar 4 if carcasses of infected reindeer and caribou are not cooked thoroughly, which may occur with traditional foods that are often eaten raw [73]. Human infections have been described in Alaska, Canada, and Siberia, with clinical signs including febrile illness, joint pain and enlargement of the liver and spleen [66].

#### Anthrax (*Bacillus anthracis*)

Anthrax is a severe infectious disease caused by the gram-positive bacterium *Bacillus anthracis*, which has a global distribution and can impact both human and animal health [66]. Anthrax was rather common in livestock in Fennoscandia until the mid-20th century, but outbreaks are now rare. The last case in Norway was recorded in 1993, but Sweden and Finland have had more recent cases [74, 75]. None have been in reindeer. Anthrax is a notifiable disease.

Anthrax spores persist in the environment for many years due to the formation of spores that are resistant to harsh conditions, including temperature, pH, pressure, chemical disinfectants, and desiccation [76, 77]. Anthrax outbreaks in livestock are generally linked to the ingestion or inhalation of *B. anthracis* spores by susceptible grazing herbivores [78]. The bacteria thrive in the aerobic environment that the infected host provides and produce several toxins that disrupt blood vessel linings, resulting in leakage of fluid that eventually leads to shock, death, and contamination of the surrounding environment near carcasses [77, 78]. Spores in the environment can then infect other animals and insects, which may serve as vectors to spread the bacteria even further [78]. Severity of disease varies between species, with severe disease (paracute or acute septicemia) generally developing in domestic and wild ruminants [66, 78].

Anthrax in reindeer is mostly known from the Siberian Taiga (also known as Siberian Plague), where large outbreaks have occurred during the summer following thawing and exposure of old anthrax burial sites [79]. One of the most recent outbreaks during the summer of 2016 resulted in the death of more than 2000 reindeer and the hospitalization of 90 residents, with one boy dying [80]. Reports of outbreaks outside of Russia are scarce and there are no reports for caribou in North America [66, 77].

#### Necrobacillosis (*Fusobacterium necrophorum*)

Necrobacillosis is a disease caused by the bacterium *Fusobacterium necrophorum*, an obligate anaerobic rod that is considered to be a normal part of the microbiome of the rumen in reindeer [81]. In general, there are two subspiecies found in ruminants, including subsp. *funduliforme* and subsp. *necrophorum*, the latter producing more toxins which makes it more pathogenic than *funduliforme* [82]. In reindeer, necrobacillosis appears in two clinical forms [66]. The first is digital necrobacillosis, an infection in the digits and distal feet, which was rather common in herds of semi-domesticated reindeer until the mid-20th century when the practice of milking reindeer ceased [83]. Bovine digital necrobacillosis is associated with wet grounds [82]. Similarly, digital necrobacillosis outbreaks in Norwegian wild reindeer have been found to occur during years with unusually high precipitation and air temperatures [81]. Infections are almost always associated with warm seasons [66].

Though digital necrobacillosis has recently been diagnosed in wild reindeer in Norway [81], this form of the disease is no longer seen in semi-domesticated reindeer. However, oral necrobacillosis, with severe lesions in the oral mucosa, oesophagus and rumen, has become a more common form of the disease that causes larger disease outbreaks [21]. New herding practices, such as supplementary feeding and calving in fenced areas, may provide opportunities for increased transmission of necrobacillosis as animals spend more time in areas heavily contaminated with faeces shed by infected animals [66].

The most common manifestation of disease in humans associated with *F. necrophorum* is upper respiratory infections, or more invasive forms with abscess formation in the peritonsillar area and thrombosis affecting the internal jugular vein (known as classic Lemièrre’s syndrome), which can eventually lead to septicaemia [84, 85]. Although this pathogen infects both animals and humans, phylogenomic studies have grouped isolates from humans differently than isolates from animals [85]. Thus, transmission from animals to humans is not obvious, and there are no reports of human infections that have been associated with transmission from reindeer.

#### Tuberculosis (*Mycobacterium bovis*)

Tuberculosis, caused by infection with mycobacteria in the *Mycobacterium tuberculosis* complex, is one of the most widespread infectious diseases worldwide. Human tuberculosis is most often associated with *M. tuberculosis*, though clinically indistinguishable zoonotic cases can occur following exposure to infected milk or by close contact with infected animals carrying *M. bovis* (via inhalation or by dressing infected carcasses) [86, 87]. Infection of various species of cervids with *M. bovis* has been reported, but *M. bovis* infections is exceedingly rare and poorly documented in reindeer [66, 88]. Experimental infection has revealed that reindeer can become infected with *M. bovis*, though lesions are less severe and less widely disseminated than those that are seen with white-tailed deer [88]. Thus, *Rangifer* are capable of contracting tuberculosis but are not considered to be a significant source of *M. bovis* exposure for human populations. A recent outbreak of *M. bovis* occurred in cattle in Norway in 2022 [89]. *Mycobacterium bovis* infection is a notifiable disease.

#### Salmonellosis (*Salmonella enterica*)

*Salmonella enterica* are gram-negative bacteria that are spread through environmental contamination by faecal shedding and have broad implications for human and animal health worldwide [90]. Salmonellosis is the second most common gastrointestinal infection in the European Union and European Economic Area (EU/EEA), with almost 66,000 verified human cases in 2022 [91]. In Norway, 757 human cases were reported in 2023, of which 243 contracted the infection in Norway [63]. Illness and outbreaks in human populations are often attributed to exposure via contaminated foods [92]. Poultry, pigs, cattle and reptiles are important reservoirs for *Salmonella* [91]. Following entry into the host by the fecal-oral route, the bacteria often become established as a subclinical infection in the ileum, cecum, and colon of animals [66].

Faeces of slaughtered reindeer have been tested across Fennoscandia, with extremely low prevalence reported (0–1%) across all countries [61, 93, 94]. The only exception is in a research herd from Finland that was kept in a restricted area, where the prevalence exceeded 10% in 1997–1998 [93]. However, *Salmonella* has not been detected since the 1990s in herds from Finland [66]. These species of bacteria are easily spread during close contact between individuals, so the movement towards supplementary feeding and crowding of reindeer for winter feeding may increase the potential for transmission [62]. Salmonellosis is a notifiable disease (food, feed and animals).

### Viral zoonoses

#### Contagious ecthyma in reindeer (Parapoxvirus)

Parapoxvirus cause proliferative processes in the skin and mucosal membranes in reindeer, like contagious ecthyma in sheep and goats. Parapoxvirus is a genus in the subfamily *Chordopoxvirinae*, family *Poxviridae*. Orf virus (ORFV), the prototype of the genus, is found in sheep and goats worldwide and causes similar lesions in a wide range of wildlife species, in addition to being a zoonosis [95, 96]. Contagious ecthyma has been reported in semi-domesticated reindeer under natural herding conditions in Finland [97], Sweden [98], and Norway [99]. In contrast to Sweden and Norway, where only a few outbreaks have been recorded, Finland has experienced serious outbreaks almost continuously since 1992 [100]. Lesions usually start as a papule that progress to a pustule and then a cauliflower-like proliferative lesion, usually at the muco-cutaneous junction of the mouth and nostrils and often involving the oral mucosa. The lesions are vulnerable to bleeding and secondary bacterial infections and animals may be unable to feed.

Whereas ORFV has repeatedly caused contagious ecthyma in semi-domesticated reindeer in Fennoscandia, more recent outbreaks in Finland, with similar pathology, have been caused by Pseudocowpoxvirus (PCPV), a close virus relative to ORFV that is associated with cattle [100]. As with sheep and farmers, Parapoxvirus can be transferred to humans during handling of live or slaughtered reindeer, causing painful skin lesions [101]. This makes Parapoxvirus one of few zoonotic pathogens that are transmitted through contact, making it very relevant for slaughterhouse workers. There is no specific treatment or vaccination for humans or reindeer. The disease is probably underreported, especially when it occurs with less severity and in a small number of animals.

#### Rabies (rabies virus)

Reindeer and caribou are not reservoir hosts for rabies, but may be affected as accidental hosts during epizootics in reservoir species in their ecosystems, such as the arctic fox (*Vulpes lagopus*) in the Arctic region and other carnivore species (e.g., red fox, *Vulpes vulpes*; raccon dog, *Nyctereutes procyonoides*; raccoon, *Procyon lotor*; skunks, family *Mephitidae*; wolf, *Canis lupus*; wolverine, *Gulo gulo*, and others) in sub-arctic regions [102, 103]. Rabies is caused by rabies virus, which belongs to genus *Lyssavirus* in the *Rhabdoviridae* family. Bats (order chiroptera) are maintenance hosts for many lyssaviruses, whereas carnivores (order carnivora) are the maintenance hosts for rabies virus (RABV). RABV cause acute progressive encephalomyelitis (rabies) in mammals and is transmitted between hosts by bites, but also by contamination of broken skin or mucosal membranes with saliva containing infective virus. The virus enters nerve tissues at the inoculation site and travels to the central nervous system (CNS). After proliferation in the brain, the virus causes encephalomyelitis and is shed in saliva.

Clinical signs of rabies in reindeer consist of confusion, weakness, incoordination, head weaving, paralysis in the hind limbs and sometimes aggression [104]. Eight clinical cases of rabies in Svalbard reindeer (*Rangifer tarandus platyrhynchus*) were diagnosed in September-October 2011 during an outbreak in the arctic fox population at Spitsbergen [105], the largest island of the high Arctic Svalbard archipelago. Since reindeer often develop lameness and paralysis, rabies is often more easily recognized in this species than in foxes [106]. Although no clinical human cases of rabies were recorded during this outbreak, about 200 animals were shot during the regular hunt and meat had already been prepared and consumed prior to detection of the virus, which could have allowed for exposure to RABV [106]. Ingestion of properly cooked or even raw meat is not generally regarded as a source of infection for people [106]. However, in a study of 1839 clinical and fatal human cases of rabies in the Philippines, 21 cases (1.14%) seem to have occurred after eating raw dog meat [107]. This may have implications for consumption of reindeer meat during an outbreak, but also for the spread of rabies during an epizootic by scavengers.

Rabies is efficiently prevented by vaccination. Vaccination trials with reindeer have been conducted, concluding that a double dose injected with an interval of 30 days may provide immunological protection for up to two years, which may be a feasible vaccine regime for semi-domesticated reindeer in rabies endemic regions [103]. Rabies is a notifiable disease.

.

### Chronic wasting disease (prions)

Chronic wasting disease (CWD) is a transmissible spongiform encephalopathy (TSE), a progressive and fatal neurodegenerative disorder affecting cervids [108]. CWD is caused by prions, which are aggregates of misfolded cell surface proteins encoded by the *PRNP* gene. Misfolded proteins, called PrP^Sc^ (termed after the scrapie prion in sheep) interact with normal prion proteins (PrP^C^) and convert them into three-dimensional copies of the misfolded protein. These misfolded prion proteins create aggregates (prions) which can have neurotoxic properties as they are more resistant to breakdown and accumulate throughout the body, especially in nerve and lymphoid tissues [109].

CWD affects the central nervous system (CNS), which is reflected by clinical symptoms like abnormal behaviour, ataxia, excessive salivation, loss of appetite, polydipsia/polyuria, teeth grinding, ear drop, and standing with a lowered head position [110]. The disease was identified for the first time in 1967 in a captive mule deer in Colorado, USA [111]. Since then, CWD has been found in 30 states in the USA and four provinces in Canada and has been identified in several wild and captive deer species. CWD was diagnosed for the first time in a wild reindeer (*R. t. tarandus*) in Norway in 2016 [110] and is a notifiable disease.

Prion diseases exist in a range of animal species and humans, but CWD is one of the few TSEs that can be transmitted horizontally between individuals and between different cervid species [109]. A new variant of Creutzfeld Jacob Disease (CJD), called vCJD, occurred in UK in 1996, representing a new zoonotic form of a human prion disease that appeared after human exposure to a prion disease in cattle (bovine spongiform encephalopathy BSE) [112]. In North America, no human cases of TSE have been associated with CWD despite the detection of CWD in several cervid species over 50–60 years [113].

In Norway, the prion strains for CWD that have appeared in reindeer differ from those in other cervid species. The development of different CWD strains may represent alterations in transmission properties affecting the ability to cross species barriers [113], such as transmission to accidental hosts [109]. Thus, it is challenging to conclude that the zoonotic potential of CWD in reindeer is negligible, suggesting that prion-affected reindeer should not be used for human consumption [113, 114]. If CWD is introduced to semi-domesticated reindeer in Fennoscandia, it may have dramatic consequences for the reindeer herding industry. This includes the epidemiology of CWD and its impact on practical herding conditions [115] along with socio-cultural and political consequences [116].

### Potentially zoonotic infections in reindeer

In addition to the zoonotic pathogens mentioned above and potential food-borne pathogens (e.g., *Listeria* spp., *Salmonellae*, *Yersinia* spp. and STEC), there are several publications that report potential zoonotic agents in reindeer. These reports are based on evidence of exposure, such as the detection of specific antibodies in reindeer blood or by the isolation or detection of pathogen DNA or RNA in reindeer tissues. One method that has contributed to this is next generation sequencing (NGS) or whole genome sequencing (WGS), detecting and identifying genomes of infectious agents, often without knowing or expecting their presence. This is in contrast to PCR, where it is necessary to target a pathogen by using specific and tailored primers (i.e., you need to know what you are searching for). This has contributed to a broadened view of the circulation of well known (and sometimes unknown) pathogens without necessarily causing disease. In this section, we will review a few of these reports.

#### *Pasteurella multocida*

*Pasteurella multocida* is an anaerobic, Gram-negative coccobacillus of the family *Pasteurellaceae*. *Pasteurella multocida* is regarded as an opportunistic pathogen residing in the upper respiratory tract of many species of pets, livestock, and wildlife. Although not verified, the bacterium is thought to be enzootic in reindeer herds. It may cause haemorrhagic septicemia and bronchopneumonia in semi-domesticated reindeer. Outbreaks have been observed, often associated with gathering, transport and slaughter, resulting in sudden mortality for a few or several hundred animals [66]. Humans are usually infected following biting, scratching, licking, or by contact with nasopharyngeal secretions from animals, such as dogs and cats. It is usually restricted to the skin, but it may develop to a general and systemic infection [117]. There are no reports of humans with pasteurellosis that have been associated with disease outbreaks in reindeer.

#### *Chlamydia pecorum*

*Chlamydia pecorum* is a Gram-negative obligate intracellular bacterium belonging to the family *Chlamydiaceae* that is associated with ocular infections and disease in a range of livestock and wildlife species. During an outbreak of infectious keratoconjunctivitis (IKC) in a reindeer herd in Sweden, *C. pecorum* was identified in swab samples from 59 of 60 animals [118]. Several species of chlamydia, such as *C. trachomatis*, *C. pneumonia* and *C. psittaci*, cause sepsis, abortions, myocarditis, pneumonia, and encephalitis in humans [119]. There is little information available on the zoonotic potential of *C. pecorum*, but a human case of *C. pecorum*-associated pneumonia was recently reported in a 51-year-old male farmer in China, assumed to have been transmitted from sheep [120]. *Chlamydia pecorum* is challenging to culture, which is likely a reason why it is an underdiagnosed pathogen in humans [120]. However, it is also now possible to identify the bacteria via PCR. During IKC outbreaks in reindeer, owners and veterinarians are cleaning the reindeer eyes and surroundings before local antibiotic treatment and should be aware of the potential hazard of being exposed to this bacterium [118].

#### Hepatitis E virus

Hepatitis E virus (HEV) is an RNA virus that belongs to the *Hepeviridae* family. The genotypes GT3 and GT4 have been found in pigs and in a wide range of wildlife species. These have been associated with human infections and hepatitis, possibly after ingestion of undercooked meat and liver, milk, or through contact with infected animals [121]. In Sweden, antibodies against HEV have been detected in moose (*Alces alces*; 27.5%, *n* = 69), wild boar (*Sus scrofa*, 15.1%, *n* = 139), roe deer (*Capreolus capreolus*, 6.7%, *n* = 30) and red deer (*Cervus elaphus*, 6.7%, *n* = 15) [122]. Phylogenetic analyses of HEV isolates suggested that some of the human cases had HEV strains similar to those from wild boar [122]. In Norway, HEV antibodies have been detected in moose (19.5%, *n* = 164), wild reindeer (23.1%, *n* = 186) [123], and semi-domesticated reindeer (15.7%, *n* = 516) [124]. Antibodies against HEV have also been found in semi-domesticated reindeer in Russia (12.1%, *n* = 191) [125]. These findings suggest that HEV is circulating in reindeer herds and wildlife populations in Fennoscandia. Since the seroprevalence was quite stable over the sampling years (Norway; 2013–2017), it seems likely that HEV, or an antigenically similar virus, is enzootic in this population [124].

In otherwise healthy humans, HEV often cause an asymptomatic or mild disease, but may cause liver symptoms and liver deficiency, dependent on the genotype of HEV and other morbidities of the infected person. Immunocompromised people, such as those with HIV or those undergoing chemotherapy, may develop severe symptoms [126, 127]. For most human cases, the source of infection remains unknown, and there have been no reports of transmission of HEV from reindeer to humans [124].

## Discussion and concluding remarks

Looking at the human population in Fennoscandia, very few people work with reindeer and are at an increased risk for zoonotic diseases obtained via direct contact during handling and slaughter. The average consumer eats only a few hundred grams of reindeer meat annually, but reindeer meat and products thereof constitute a large part of the meat intake for reindeer herders and their local communities at large. This is also the case for Indigenous populations in Canada and Alaska’s Arctic regions, being highly reliant on caribou. Caribou meat is one of the most consumed country foods in the central Canadian Arctic [128], and Canadian Indigenous groups are at an increased risk of foodborne illnesses obtained from wildlife when compared to other citizens [129].

Methods of preparing meat without heat treatment, such as drying, smoking, curing, or raw consumption with minimal heat-treatment, can be found in many Arctic and Sub-Arctic people’s cultures, including Fennoscandia and North America, which can dramatically impact pathogen transmission [129–131]. For example, the parasite *T. gondii* relies on transmission following the consumption of tissue cysts in raw or undercooked meat. However, heating at ≥ 60 °C or freezing meat for periods ≥ two days at − 20 °C kills tissue cysts [132]. Thus, successful transmission depends on the method that is used to prepare the meat along with the method of storage; i.e., meat that is frozen for prolonged periods prior to consumption may prevent transmission of certain pathogens. Though omitting heat treatment when preparing reindeer meat could lead to increased potential for pathogen transmission, it is important to highlight that many of these pathogens are exceedingly rare and likely do not justify the abandonment of traditional and cultural practices [131].

Our discussion so far highlights the importance of a One Health approach when considering zoonotic risk from reindeer. Traditional reindeer herding in Fennoscandia requires a careful balance between pastureland, animals, and humans. When an imbalance occurs between any of these three factors, it can impact the transmission of pathogens either directly or indirectly. A clear example of this is when increasing temperatures and precipitation (rain-on-snow events) create ice layers and locked pastures that reduce the availability of forage for reindeer [133]. As reindeer herding is entirely reliant on the sustainable use of pastureland, the presence of thicker ice crusts drives more herders towards the use of supplementary feeding, which can increase the frequency of disease outbreaks in herds via the use of shared confined spaces [134, 135]. This has already been linked to disease outbreaks, including infectious keratoconjunctivitis (Alphaherpesvirus; CvHV2) and necrobacillosis (*F. necrophorum*). In addition, increased stress and challenges with food availability have a direct impact on reproductive success and calf fitness, which may also exacerbate comorbidities with infectious agents and impact meat productivity [135].

Increased demand for farmed fields also ensures shared use of pastureland between livestock and semi-domesticated reindeer, which can facilitate interspecies transmission of pathogens. A classic example of this occurs with the parapoxviruses (such as ORFV) that can cause contagious ecthyma in sheep, goats, and reindeer. Intraspecies competition for land also occurs between wild and semi-domesticated reindeer in Norway. For example, in northern Norway, wild herd populations dwindled alongside the explosion in herd size for semi-domesticated reindeer during the drive towards pastoralism in the 17th to 19th centuries [19]. This decline in wild reindeer herds during co-existence with large semi-domesticated herds likely reflects increased competition for resources. Overlapping use of land may also drive intraspecies transmission of infectious agents, such as CWD, which has only been detected in wild herds so far [110]. As both the environment and use of pastureland continue to evolve, continued surveillance for pathogens that can impact human and reindeer health should be prioritized.

Though the distribution and frequency of zoonotic pathogens in semi-domesticated reindeer herds is likely to change in the future, the current information presented in this review – the presence and prevalence of potential zoonotic pathogens – must be interpreted within the appropriate context. In spite of the growing practice of supplementary feeding, most semi-domesticated reindeer are year-around free-ranging, utilizing remote pasture resources over large areas. Even with inspection with motorized vehicles during summer and winter, the use of collars with satellite driven Global Positioning Systems (GPS) and active herding and transition between seasonal pastures, the loss of reindeer due to predators, starvation, road and train kills and other causes may be significant [7]. Since reindeer carcasses are scavenged by many species, lost animals are seldom available. If carcasses are found, they are often in a state that is not feasible for pathological investigations [136]. Furthermore, reindeer herds are seldom visited by a veterinarian. In a questionnaire among reindeer herders in Norway and Sweden, focussing on the disease infectious keratoconjunctivitis (IKC), only 7% (of 57 responders) answered that they would contact a veterinarian in case of an IKC disease outbreak [137].

This indicates that most of the information on infectious diseases in semi-domesticated reindeer is based on the period of the year when animal blood and tissues are available, i.e., during the slaughter season, through PMI and sample collection for research. Thus, many of the reports are dealing with a narrow time window, referring to the period between late fall and February. In addition, these investigations are based on animals that are suited for slaughter – animals that are too skinny or show signs of discomfort or disease will (should) not be transported or slaughtered.

There are additional reasons for why infectious diseases may not be detected or registered. Most of the reindeer that are slaughtered are calves, about 70–77% in Norway, Sweden and Finland [7–9]. This could partly explain the good health status of many of the animals in the studies that have been reviewed, which have often been based on slaughtered animals. Young animals typically have less time to be exposed to infectious diseases. This is particularly relevant for serological studies, which detect the presence of antibodies, revealing previous exposure to an infectious agent, and not generally active infection or shedding of a pathogen. Thus, long-lived animals may be more likely to be positive on serological tests, depending on how long the antibody response lasts and is detectable after an infection, which varies between pathogens. Also, since PMI in slaughterhouses are conducted with the aim of securing good animal welfare and food safety, and generally is not including specific investigations for identification and characterization of potential pathogens, many of the zoonotic infections included in this review could remain unnoticed on the slaughterhouse line. Finally, molecular detection of pathogen DNA or RNA within the tissues and/or fluids of animals relies on the presence of an active infection. The detection of the pathogen may be more or less likely depending on the time of sample collection. Again, this usually coincides with routine or research-based investigations that are often conducted during the slaughter period in the winter months. All these factors may have influenced the presence and prevalence of pathogens in semi-domesticated reindeer herds reported in this review.

From the review conducted, it can be concluded that the zoonotic threat from close contact with reindeer or the consumption of reindeer meat and products thereof currently is of restricted magnitude. However, as the balance between environment (pastureland), animals and people continue to shift, more effort is needed to establish appropriate surveillance measures to collect health and disease parameters for herds across Fennoscandia, as well as the circumpolar north. As reindeer are a keystone species in Arctic and Sub-Arctic environments and represent a strong cultural heritage across much of the circumpolar north, these efforts are imperative for people who live off the land as they face imminent changes in northern ecosystems.

## Data Availability

Not applicable.

## Abbreviations

BSE: Bovine spongiform encephalopathy
CNS: Central Nervous System (brain and spinal cord)
CvHV2: Cervid herpesvirus 2
CJD: Creutzfeldt Jacob Disease
CJDv: Variant Creutzfeldt Jacob Disease
CWD: Chronic Wasting Disease (a prion disease in reindeer and other cervids)
DNA: Deoxyribonucleic acid
EEA: European Economic Area (EU/EEA)
EU: European Union
FFA: Finish Food Authority
GPS: Global Positioning Systems
HEV: Hepatitis E virus
HIV: Human Immunodeficiency Virus
IKC: Infectious keratoconjunctivitis
NFSA: Norwegian Food Safety Authority
NGS: Next generation sequencing
ORFV: Orf virus, the type species of the Parapoxvirus genus
OMI: Ophthalmomyiasis interna
PCPV: Pseudocowpoxvirus
PMI: *post mortem* inspection; slaughterhouse meat inspection
POP: Persistent organic pollutant
PRNP: Prion protein gene, encoding the prion protein
PrP^C^: The physiological cellular prion protein
PrP^Sc^: Prion protein scrapie; an abnormal, pathogenic, and infectious conformer
RABV: Rabies virus, genus *Lyssavirus*
RNA: Ribonucelic acid
SFA: Swedish Food Agency
STEC: Shiga-toxin producing *Escherichia coli*
TSE: Transmissible Spongiform Encephalopathy
WGS: Whole Genome Sequencing
WHO: World Health Organization
Zoonosis: An infectious disease that is naturally transmissible from vertebrate animals to humans
